# Development and evaluation of male-only strains of the Australian sheep blowfly, *Lucilia cuprina*

**DOI:** 10.1186/1471-2156-15-S2-S3

**Published:** 2014-12-01

**Authors:** Maxwell J Scott

**Affiliations:** 1 Department of Entomology, North Carolina State University, Campus Box 7613, Raleigh, NC, 27695-7613, USA

## Abstract

The Australian sheep blowfly *Lucilia cuprina *(Wiedemann) is a major pest of sheep in Australia and New Zealand. From the 1960s to the 1980s there was a major effort to develop "field female killing" or FFK strains of *L. cuprina *that could be used for a cost-effective genetic control program. The FFK strains carried eye color mutations that were lethal to females in the field but not under conditions in the mass rearing facility. Males did not die in the field as normal copies of the eye color genes had been translocated to the Y chromosome and an autosome. Although the FFK strains showed some promise in field tests, a genetic control program in mainland Australia was never implemented for several reasons including instability of the FFK strains during mass rearing. A stable transgenic strain of *L. cuprina *that carried one or more dominant repressible female lethal genes offered the potential for efficient genetic control of blowfly populations. Here I review our research on tetracycline-repressible female lethal genetic systems, *Lucilia *germ-line transformation and sex determination genes that ultimately led to the successful development of transgenic "male-only" strains of *L. cuprina*. The technology developed for *L. cuprina *should be directly transferable to other blowfly livestock pests including *L. sericata *and the New World and Old World screwworm. 29

## Background

Female *Lucilia cuprina *lay eggs on the live animal and their larval offspring cause a cutaneous myiasis (flystrike) in sheep. *L. cuprina *is the major primary strike fly (fly that causes flystrike) in Australia [1] and an increasingly significant strike fly in New Zealand [2]. Flystrike can lead to reduced wool quality and quantity and death of the animal if not treated, which largely involves the widespread use of insecticides and good farming practices. The annual economic cost of flystrike is estimated to be AUS $280 million in Australia [3] and NZ $30-40 million in New Zealand [2]. For many years there was considerable interest in developing strains of *L. cuprina *that could be used for efficient genetic control programs. These efforts were inspired by the success of the sterile insect technique (SIT) in eradicating the related New World screwworm fly (*Cochliomyia hominivorax*) from the U.S.A. [4,5]. Subsequently, New World screwworm was eradicated from all North and Central America [5]. SIT involved mass rearing of the insect, sterilization by radiation and aerial release of sterile males and females over the targeted area. As the sterile males were in a large excess (at least 9:1) over fertile males in the field, the fertile females in the area were more likely to mate with the sterile males. For genetic control of *L. cuprina *to be cost-effective in Australia, strains were developed that were predicted to be more effective at lower release ratios than a bisexual release and that did not need to be sterilized by radiation [6,7]. The latter would eliminate the need for a central large expensive mass rearing facility built around a radiation device. Instead several smaller facilities could be built which were expected to be more cost effective given the size of the Australian continent and distribution of sheep blowfly [7]. Eliminating the radiation step could also improve the fitness of the released males, as radiation does reduce the fitness of *C. hominivorax *males [8].

"Field female killing (FFK)" strains were developed that were homozygous for two autosomal recessive eye color mutations that essentially made the females blind in the field [7]. The FFK strains carried multiple chromosomal translocations involving the Y chromosome and the two autosomes that had normal copies of the eye color genes. Consequently, males were not blind and were semi-sterile, as most of their offspring would not develop due to aneuploidy. The combination of male semi-sterility and recessive female lethality was predicted to make the FFK strain more effective at population reduction than SIT, particularly at the more cost-effective low release ratios [9]. Indeed, a small island (40 km^2^) trial in 1985-1986 was successful, achieving a very high genetic death and suppressing the *L. cuprina *population to undetectable levels [7]. A subsequent larger island field trial initially had some success but ultimately failed. There were problems with mass rearing the strain, which was unstable and prone to breakdown due to recombination in males. With a decline in the wool price, the *L. cuprina *genetic control program was abandoned. Nevertheless, the concept of releasing flies that were not sterilized by radiation but carried female lethal genes had been established [10].

### Development and evaluation of tetracycline-repressible female lethal genetic systems in *Drosophila melanogaster*

The successful germ-line transformation of *D. melanogaster *in 1982 [11] opened the potential for making transgenic insect strains carrying female lethal genetic systems. As females would be needed for mass rearing, female-specific lethality needed to be repressible. In the mid 1990s, not long after establishing our laboratory at Massey University in New Zealand, we began building and testing such genetic systems in *D. melanogaster *since there were many genetic tools available for *Drosophila *engineering. Also a method for germ-line transformation of *L. cuprina *and not yet been developed.

**Figure 1:**
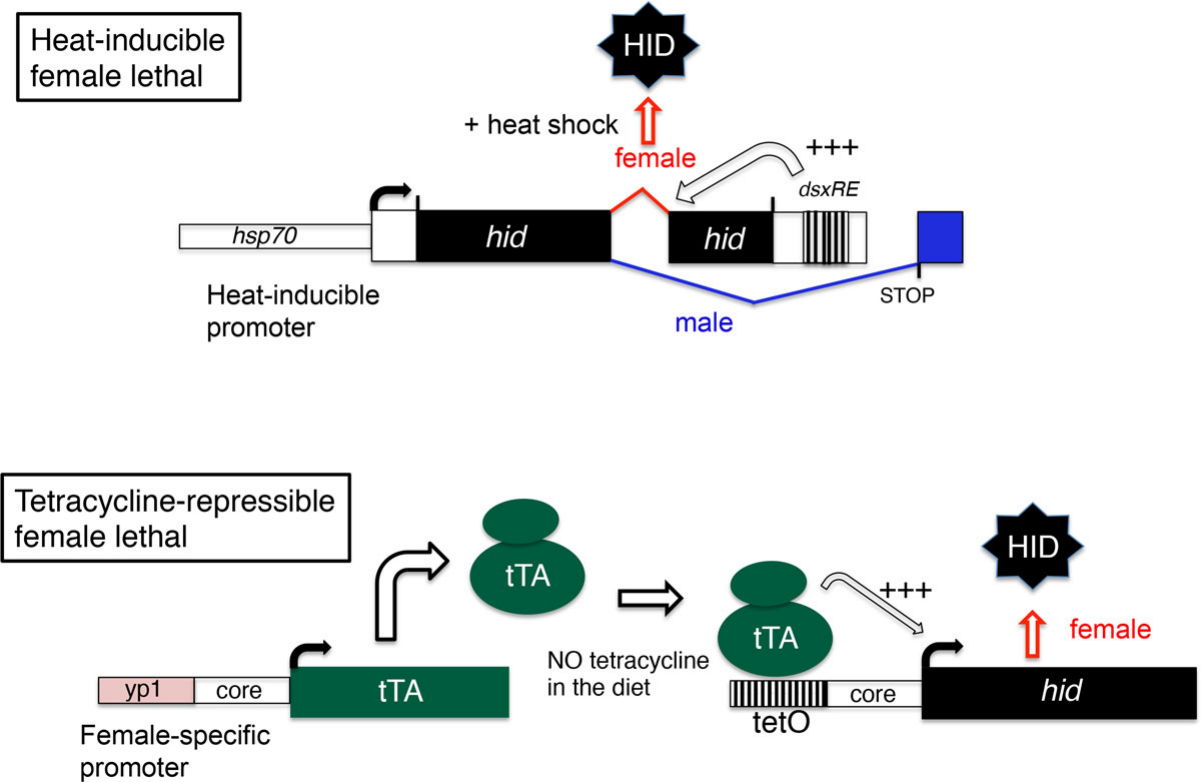
**Conditional female lethal genetic systems evaluated in *Drosophila melanogaster***. (Top) Heat inducible female lethal system. The *hid *open reading frame was interrupted with the female-specific intron from the *dsx *gene, which contains a weak splice acceptor site. Immediately 3' of *hid *was the *dsxRE*, which includes several TRA/TRA2 binding sites. Binding of TRA/TRA2 to the *dsxRE *enhances (+++) splicing of the *dsx *intron in females. Further downstream was the intron-exon from the *Dror2 *gene, which has an optimal splice acceptor site. Males were predicted to use the stronger *Dror2 *splice site whereas females would use the *dsx *acceptor site. Thus only the female *hid *transcript was predicted to encode full-length protein. (Bottom) Tetracycline-repressible female lethal system. On normal diet, expression of tTA in the female fat body of third instar larvae led to activation (+++) of *hid *gene expression and female-specific lethality. Lethality was repressed by the addition of tetracycline to the diet.

To achieve female-specific gene expression, it was apparent that this required either a gene promoter that was female-specific or an intron that was sex-specifically spliced. For the latter, we turned to the genes that are part of the well-characterized *Drosophila *sex determination regulatory pathway [12]. Transcripts from the master gene *Sex lethal*, from the *transformer *(*tra*) gene and from the *doublesex *(*dsx*) gene were all known at that time to be sex-specifically spliced. Wilkins had suggested that the genes at the bottom of the regulatory pathway, *dsx *and *fruitless*, would be more highly conserved than the master gene at the top of the pathway [13]. This hypothesis has largely proven to be correct. Consequently, we focused on using the sex-specifically spliced intron from the *D. melanogaster dsx *(*Dmdsx*) gene as we reasoned that *dsx *transcripts were also likely to be sex-specifically spliced in *L. cuprina*. Steller and colleagues had shown that widespread expression of the proapoptotic gene *head involution defective *(*hid*), also known as *Wrinkled *(*W*), led to organismal death [14]. Thus we decided to insert the *Dmdsx *intron within the *hid *gene to obtain a female-specific lethal gene. Expression was controlled with the heat inducible *hsp70 *gene promoter [15] (Figure 1). The expectation was that after a heat shock, *D. melanogaster *females would die, as only the female *hid *transcript would code for fully functional HID protein. Unfortunately, after heat shock both males and females died as in both sexes *hid *transcripts were spliced using the weak female-specific *Dmdsx *splice acceptor site. It appeared that the *hid *transcript contained a nucleotide sequence that enhanced the use of the weak female acceptor site in both sexes [15].

Fortunately, the alternative approach of using a female-specific gene promoter was more successful. A 125 bp enhancer from the *yolk protein 1 *(*yp1*) gene had been shown to drive expression of a reporter gene in female fat body [16]. This could have been used to control expression of *hid *to achieve female lethality but this system would not have been conditional. To achieve regulated expression of *hid*, we placed the tetracycline-dependent transactivator or tTA gene under the control of the *yp1 *enhancer (Figure 1). tTA is a fusion of the DNA binding domain from the *Escherichia coli *tet repressor (tetR) and the viral VP16 transcription activation domain [17]. In the presence of tetracycline, tTA does not bind to the operator (*tetO*) from the *E. coli *tetracycline-resistance operon [17]. We found that in transgenic *Drosophila, yp1-tTA *activated expression of a *tetO-lacZ *reporter gene in female fat bodies but only in the absence of tetracycline [18]. Thus we had a made a female-specific conditional expression system. The last step was to make *tetO-hid *"effector" lines and cross with yp1-tTA "drivers" (Figure 1). We found that females carrying both components died unless tetracycline was added to the diet [18]. Thus we had developed a transgenic FFK system. Unknown to us, a group in Oxford led by Luke Alphey had developed an almost identical system, which they called RIDL for "release of insect carrying dominant lethals" [19]. Modeling suggested that the RIDL system would be more efficient than SIT [19], particularly if the strains carried multiple repressible female-lethal genes that had low fitness costs [20]. It should be noted that, although there are several studies on fitness costs in transgenic RIDL strains (e.g. [21,22] and references therein), this remains to be evaluated under mass rearing and under field conditions.

### Germ-line transformation of *L. cuprina*

*Drosophila *transformation was achieved by using vectors based on the *P *transposable element. However, plasmid-based mobility assays showed that the *P *element was not functional outside of Drosophilidae [23]. Thus the first step in developing a *L. cuprina *transformation system was to identify transposable elements that were functional in *L. cuprina *embryos. Peter Atkinson and colleagues used interplasmid transposition assays to show that the *mariner *transposable element was functional in *L. cuprina *[24]. We used similar assays to show that the *Minos *and *piggyBac *transposable elements were active in *L. cuprina *embryos [25]. The next step was to identify a marker gene that could be used to identify transgenic *L. cuprina*. Initially we used eye color genes as several *L. cuprina *strains carrying eye color mutations were available, including *white *eye. However, our attempts at making transgenic *L. cuprina *using *Minos *or *piggyBac *vectors carrying the medfly *white *gene as a marker were unsuccessful [25]. An alternative to eye color genes were fluorescent protein marker genes [26]. An EGFP gene driven by the *D. melanogaster polyubiquitin *promoter (*PUbnlsEGFP*) had been successfully used to identify transgenic *D. melanogaster *and *Anastrepha suspensa *[27,28]. Using a *piggyBac *vector containing this EGFP marker gene we were successful in transforming *L. cuprina *[25]. The same vector was subsequently used to make transgenic *C. hominivorax *[29].

**Figure 2:**
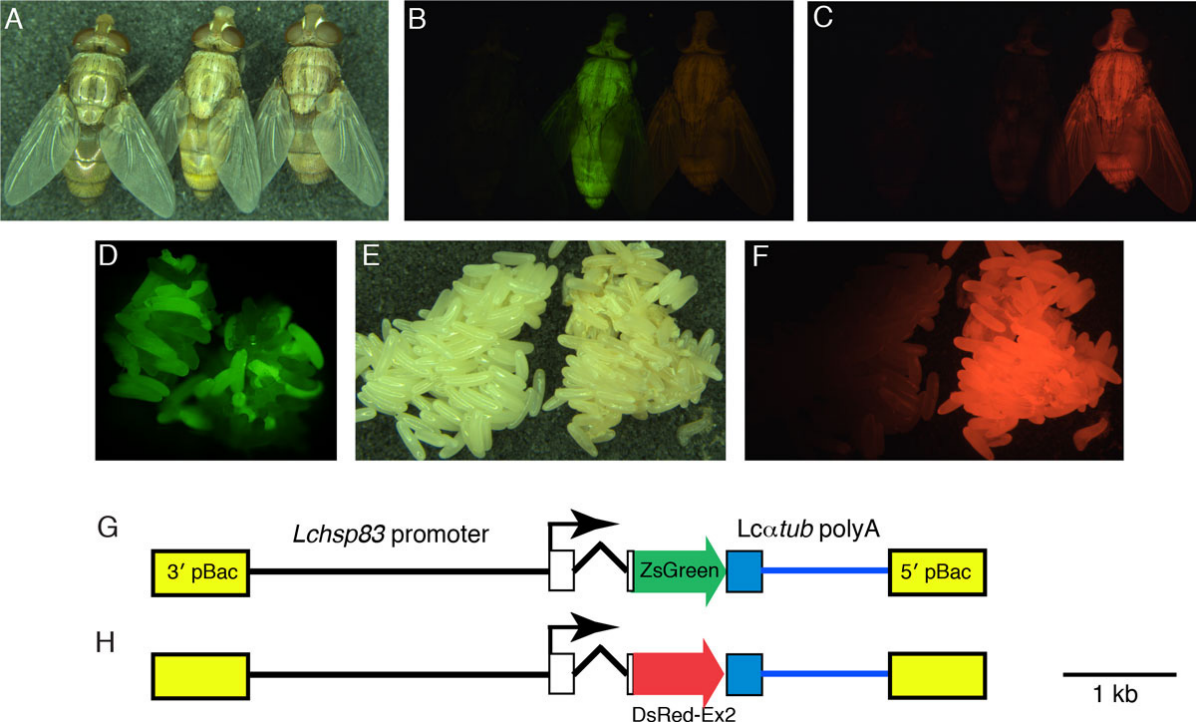
**Marker gene expression in transgenic *Lucilia cuprina***. Adult flies or embryos were observed with either white light (A,E), a long pass GFP filter set (B,D) or a DsRed filter set (C,F). (A-C) Young adults (less than 2h after eclosion) from the wild-type stock (left), a transgenic line that carries the ZsGreen marker (center) and a line that has the DsRed-Express 2 (DsRedex2) marker (right) are shown. (D) A mixture of non-transgenic and transgenic (ZsGreen marker) late stage embryos. The transgenic embryos show strong green fluorescence and are easily separated from the non-transgenic embryos. (E,F) Embryos from the wild-type stock (left) and from a transgenic line that carries the DsRedex2 marker (right). Transgenic individuals show bright green or red fluorescence in all cells from the mid-embryo to young adult stage. In older adults that have darker cuticles, fluorescence is much more difficult to detect (not shown). Consequently, we routinely screen for transgenic individuals at the embryo and larval stages. (G,H) Schematic representations of the pB[Lchsp83-ZsGreen] and pB[Lchsp83-DsRedex2] transformation vectors The vectors contains the 5' flanking DNA, first exon and first intron from the *Lchsp83 *gene.

Although we had succeeded in making transgenic *L. cuprina*, the overall efficiency was low and most experiments did not produce any transgenic flies. One reason for the low efficiency appeared to be because the *D. melanogaster polyubiquitin *promoter was weakly active in *L. cuprina *(and *C. hominivorax*), which made it difficult to distinguish transgenic from non-transgenic larvae. Consequently, we isolated the strong *hsp83 *gene promoter from *L. cuprina *[30]. In *D. melanogaster*, the *hsp83 *promoter has a high basal activity in all cells and is active in the germ-line [31]. The *Lchsp83 *promoter was used to make more strongly expressed fluorescent protein marker genes and *piggyBac *transposase helper. These modifications led to an efficient and reliable method for germ-line transformation of *L. cuprina *[32]. Transgenic individuals are readily identified at the late embryo or larval stages and show strong whole body fluorescence (Figure 2).

### Genes that are sex-specifically expressed in *L. cuprina*

In order to develop transgenic *L. cuprina *carrying the tetracycline-repressible female lethal system that was successful in *D. melanogaster*, we isolated a *L. cuprina *yolk protein gene promoter (*LcypA*) [33]. *L. cuprina *that carried an *LcypA-lacZ *transgene showed high levels of β-galactosidase in adult females, but only after a protein meal. Thus, although the *LcypA *promoter was female-specific, it was active at too late a stage for a FK system [33].

**Figure 3:**
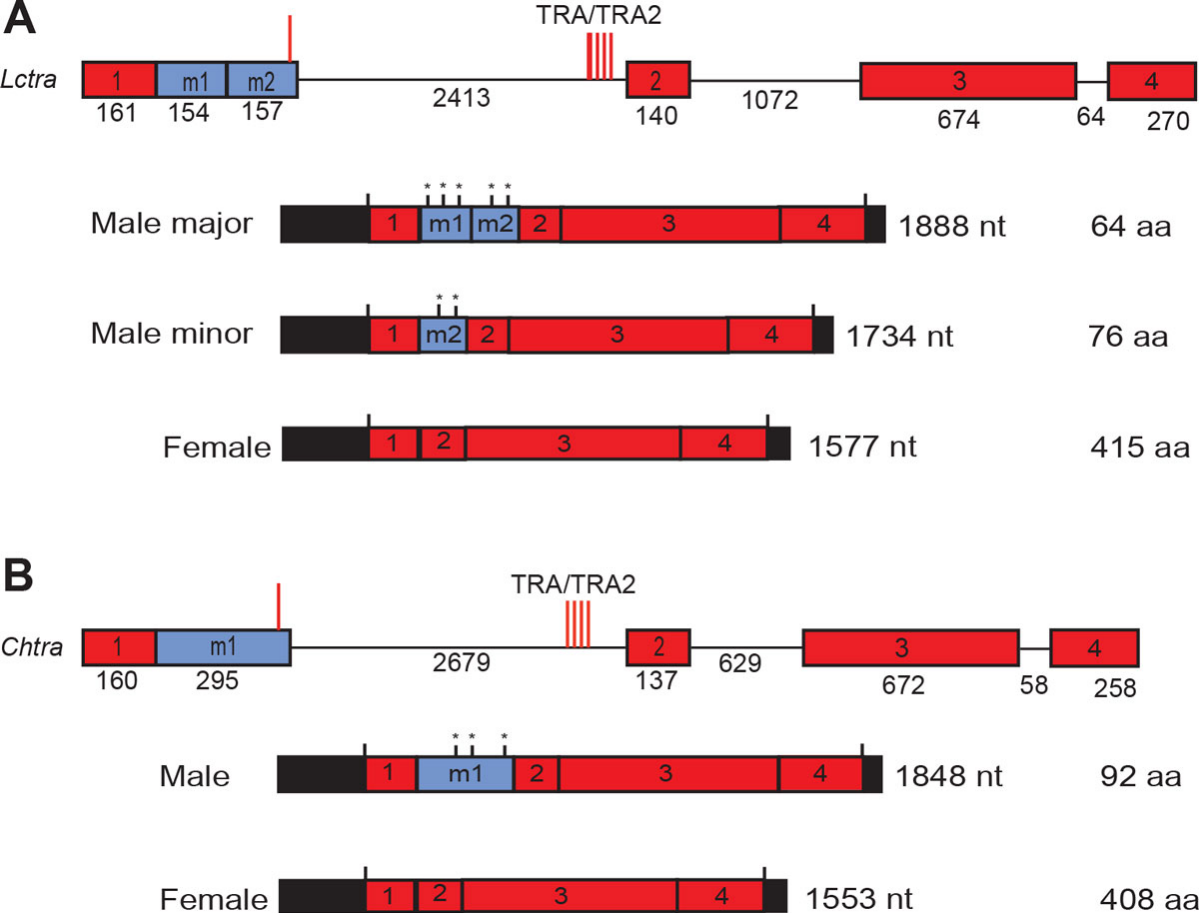
**Comparison of the genomic organization and sex-specific transcripts of the *L. cuprina *and *C. hominivorax transformer *genes**. The diagrams represents the *Lctra *(A) and *Chtra *genes (B). The exons are shown as square boxes, with exons in red representing common exons to both female and male mRNAs. Exons m1 and m2 in blue represent male specific exons. Introns are represented by solid lines. Exon and intron sizes are indicated. Red vertical lines represent the position of putative TRA/TRA2 binding sites within the male exon and the first intron. The male and female transcripts are shown below the genes. The 5' and 3' untranslated regions are represented by black boxes. Vertical black lines mark translational start and stop sites and in-frame translational stop sites in the male exons are marked with asterisks. The predicted lengths of the proteins encoded by the transcripts are indicated. *Lctra *and *Chtra *have a very similar exon-intron arrangement and sex-specific splicing pattern. Modified from [37].

An alternative approach was to return to using introns from sex-specifically spliced transcripts to achieve female-specific gene expression at an earlier stage of development. Giuseppe Saccone and colleagues had shown that in the Mediterranean fruit fly, *tra *transcripts are sex-specifically spliced and splicing is autoregulated [34]. Moreover, RNAi-mediated knockdown of *tra *expression led to the transformation of XX individuals to males. This suggested that it could be possible to make a repressible female-male transformation system, which modeling suggests could be a very effective means for population control [35]. However, isolation of the *L. cuprina tra *gene proved to be a formidable challenge as the *tra *gene was poorly conserved between *Drosophila *and medfly. A fragment of the *L. cuprina tra *gene was isolated using PCR with degenerate primer pairs based on conserved amino acid motifs [36]. Subsequent analysis found that only the female *tra *transcript codes for TRA protein. The major male transcript includes an additional exon with several in-frame stop codons (Figure 3). As in *C. capitata*, the presence of multiple predicted TRA/TRA2 binding sites within the sex-specifically spliced first intron strongly suggested that splicing was autoregulated. The *tra *gene was shown to be essential for female development as injection of *tra *double-stranded RNA into the posterior end of embryos led to the development of XX adults with male genitalia. More recently we have isolated the *tra *gene from *L. sericata, C. hominivorax *and *C. macellaria *[37]. The overall organization of the *tra *genes from the four blow fly species is remarkably conserved, with a similar exon-intron arrangement and relative location of TRA/TRA2 binding sites (Figure 3). Lastly, we also isolated the *L. cuprina dsx *gene and showed that *dsx *transcripts are sex-specifically spliced as in *Drosophila *and housefly [38]. The presence of eight TRA/TRA2 sites in the female exon strongly suggested that *dsx *splicing in female is regulated by TRA as in *Drosophila*.

### Development of transgenic "male-only" *L. cuprina *lines

**Figure 4:**
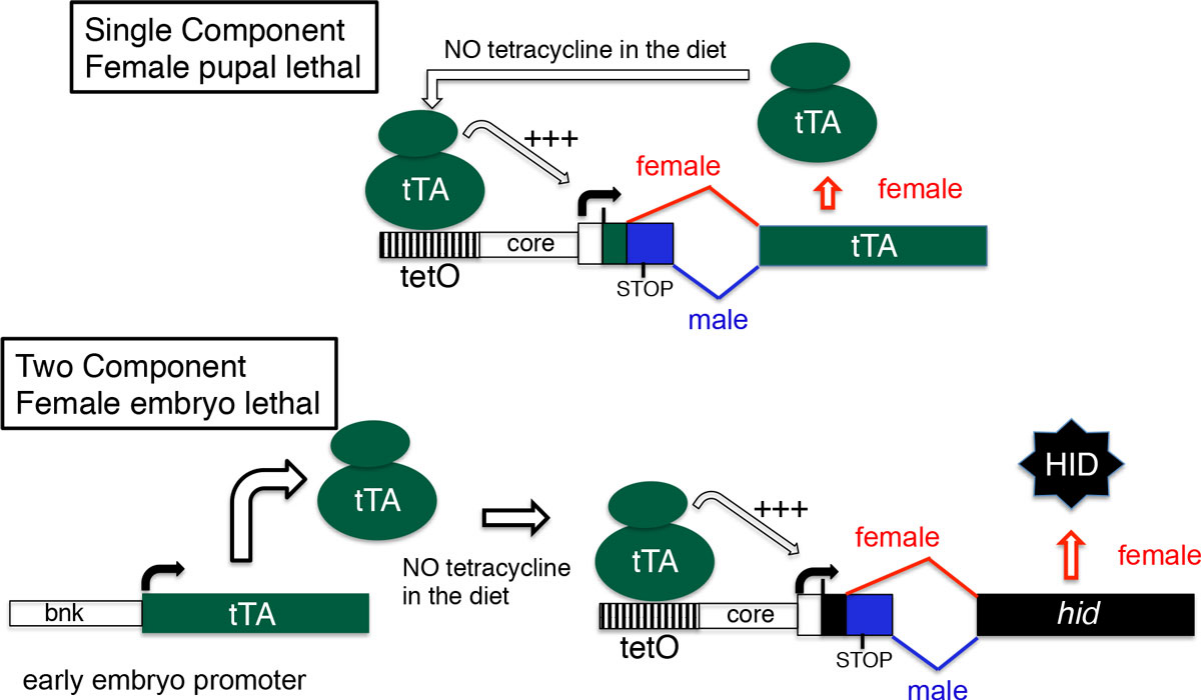
**Tetracycline-repressible female lethal genetic systems for *Lucilia ******cuprina***. In the single component system [39], *tTA *expression is auto-regulated as the promoter contains 21 copies of the tTA binding site (*tetO*) upstream of the core promoter from the *Lchsp70 *gene [40]. The *Chtra *intron is inserted within the tTA coding region. As a consequence, only female overexpress tTA due to sex-specific splicing of tTA transcripts. Binding of tTA to tetO results in activation (+++) of tTA gene expression. High female lethality was observed in most transgenic lines when reared on diet that lacks tetracycline [40]. In the two-component system [41,42], which is under development for *L. cuprina*, tTA expression is controlled by a promoter from a cellularization gene (e.g. *bnk*), which is most active in early embryos. tTA activates (+++) expression of *hid*, but only the female *hid *transcript encodes HID protein due to sex-specific splicing of the *Chtra *first intron.

Moving to the U.S.A., the focus of the lab shifted to developing a transgenic male-only strain of the New World screwworm. However, since it was not possible to work with *C. hominivorax *in the U.S.A., we continued to work with *L. cuprina *(using a North American strain) as it is a close relative. Initially, we built a one-component system (Figure 4), based on the system developed by Luke Alphey and colleagues at Oxitec [39]. They found that overexpression of an auto-regulated tTA was lethal (usually pupal stage) but repressible by tetracycline. The lethality was thought to be due to a general interference with gene expression or "transcriptional squelching". The lethality can be made female-specific by incorporating the sex-specifically spliced *tra *intron into the tTA gene [39]. The appeal of making this system was that we had all of the necessary components, including the first intron from the *C. hominivorax tra *gene and an efficient transformation system. A disadvantage was that the late lethal period would not lead to any appreciable savings in the costs of diet for mass rearing. The initial system, FL3, used the core promoter from the *D. melanogaster hsp70 *gene and multiple copies of the tTA binding site (tetO) upstream of the core promoter. Two transgenic *L. cuprina *FL3 lines were made. In one line, over 99% of homozygous females died on diet that lacked tetracycline [40]. However, there was no decrease in female viability in heterozygous females. Further, homozygous females in the second line were fully viable on standard diet. Interestingly, in transgenic *Drosophila *FL3 lines, 100% of females died on diet lacking tetracycline. Thus the *C. hominivorax tra *intron was correctly sex-specifically spliced in *D. melanogaster*. However, it appeared that the system was not optimal for high levels of tTA expression in *L. cuprina*. We considered that expression could be improved by replacing the *Drosophila *core promoter with the core promoter from a *L. cuprina hsp70 *gene [30]. Two gene constructs were made, FL11 and FL12, which differ slightly in the length of the core promoter. In three of five FL11 lines and two of three FL12 lines, 100% of homozygous females die on diet that lacks tetracycline [40]. Moreover, for three of the lines female lethality is dominant, with one copy of the transgene sufficient for 100% female lethality. Thus these lines could potentially be used for a RIDL genetic control program [40]. Surprisingly, the sexes could be reliably sorted by fluorescence or color at larval stages as females that overexpress tTA also overexpress the linked marker gene. It would appear that tTA bound to tetO is either directly enhancing expression of the *Lshsp83 *promoter that drives the red fluorescent protein marker, or there is an indirect effect through changes to the structure of the local chromatin domain. Female larvae make so much of the marker protein that they show a crimson color under white light, easily distinguished from male larvae that have a very pale pink color.

To achieve female lethality at an earlier stage of development, we have also been working on a two-component genetic system (Figure 4). A similar system was used to develop *A. suspensa *and *C. capitata *transgenic embryonic sexing strains [41,42]. In this system a promoter from a cellularization gene (e.g. *sry-*α *bnk, slam, nullo*) that is most active in early embryos is used to drive expression of tTA. The effector is a *tetO-hid *construct that contains the sex-specifically spliced *tra *intron inserted in the *hid *gene. Thus in the absence of tetracycline, tTA is expressed in the early embryo and activates transcription of *hid*, but only female embryos die as only the female transcript codes for HID protein. To make transgenic embryonic sexing strains of *L. cuprina*, promoters from *Lucilia *cellularization genes and *Lucilia *cell death genes were required. Transcripts for the *L. sericata bnk, rpr *and *hid *genes were identified in RNA-seq libraries [43]. The *L. sericata bnk *promoter was isolated and shown to be active in transgenic *L. cuprina *embryos using a GFP reporter gene [44]. Expression of the *L. sericata rpr *and *hid *genes in transgenic *Drosophila *induced widespread apoptosis, with *Lshid *being particularly effective at inducing organismal death [44]. Thus with the required components on hand, we can proceed with assembling the gene constructs necessary to make transgenic sexing strains of *L. cuprina *where females die during embryogenesis.

## Conclusion

Transgenic sexing strains of *L. cuprina *have been developed that carry a tetracycline -repressible female lethal genetic system. The successful development of these strains built upon prior research on female-lethal genetic systems in *D. melanogaster, Lucilia *germ-line transformation and sex determination genes in *Lucilia*. In the near future development of transgenic *L. cuprina *embryonic sexing strains should be possible as the required components have been isolated from *Lucilia *and shown to be functional. If the transgenic sexing strains were to be used for a genetic control program in Australia (or New Zealand), the next step would be to introgress the transgene into a more appropriate genetic background by crossing with a field strain from Australia. Cage studies would then be performed to evaluate the ability of the strains to suppress a population. The strains could be used for conventional SIT or a fertile release program. However, if fertile males are released, the male larval offspring will feed on tissue of live sheep, causing damage that may not be acceptable to growers. The systems that have been developed for *L. cuprina *should be directly portable to other blowfly livestock pests including the New World and Old World screwworm. We are actively working on making and evaluating transgenic sexing strains of New World screwworm (*C. hominivorax*).

## Competing interests

The author declares that they have no competing interests.

## Authors' contributions

Text and figures were prepared by the author.
